# Obituary

**Published:** 1879-10

**Authors:** 


### OBITUARY.

Died, July 10th, 1879, at London Ohio, of cancer of the stomach, Dr. Wm. H. Carter, in the forty-fifth year of his age.

Dr. Carter was a native of Lancaster County, Virginia, April 28, 1835. He attended one course of lectures at the Baltimore Dental College, 1865–6, practiced a short time in Cumberland, Md., after which he removed to, and located in London, Ohio, in 1869.

He was enthusiastic, and industrious in his profession.

He was a gentleman, and well esteemed by all who knew him. The profession in Ohio can illy afford to spare such men from its ranks.

